# Implementing Service-Learning Programs in Physical Education; Teacher Education as Teaching and Learning Models for All the Agents Involved: A Systematic Review

**DOI:** 10.3390/ijerph18020669

**Published:** 2021-01-14

**Authors:** Raquel Pérez-Ordás, Alberto Nuviala, Alberto Grao-Cruces, Antonio Fernández-Martínez

**Affiliations:** 1 Faculty of Human Sciences and Education, University of Zaragoza, Valentín Carderera, 4, 22003 Huesca, Spain; rpordas@unizar.es; 2 Department of Sports and Computer Science, Pablo de Olavide University, Crta. de Utrera Km1, 41013 Seville, Spain; anuvnuv@upo.es; 3 Department of Physical Education, Faculty of Education Sciences, University of Cádiz, Avda. República Saharaui s/n, Campus de Puerto Real, 11519 Cádiz, Spain; alberto.grao@uca.es

**Keywords:** physical education, community service, methodology, pre-service teacher

## Abstract

Service-learning (SL) is the subject of a growing number of studies and is becoming increasingly popular in physical education teacher education (PETE) programs. The objective of this study was to conduct a systematic review of the implementation of SL programs with PETE students. The databases used were Web of Science, SPORTDiscus (EBSCO), and SCOPUS. Articles were selected on the basis of the following criteria: (a) published in a peer-reviewed journal; (b) covers the use of SL programs with PETE students; (c) relates to physical education or physical activity programs; (d) availability of a full-text version in English and/or Spanish. Thirty-two articles met the inclusion criteria. Two types of findings were observed: firstly, findings relating to the study characteristics and objectives and, secondly, recommendations for improvement of this type of intervention. The objectives of the different studies focused on (a) the impact of the SL methodology on PETE students’ professional, social, and personal skills; (b) its impact on the community; (c) analysis of the effectiveness and quality of the programs. All but two studies analyzed the impact of SL on PETE, while only four analyzed community participants and only three analyzed the quality of the SL program. Recommendations for improving SL programs used with PETE students included: all stakeholders, e.g., students and community participants, should be studied and coordinated; the quality of the programs should be assessed, as studying the effectiveness of SL programs could help to attain the objectives of both students and the community; mixed methods should be used; and intervention implementation periods should be extended to provide more objective, controlled measurements.

## 1. Introduction

Service-learning (SL) has been defined in multiple ways. The common thread running through these definitions is that SL is a methodological strategy that involves a program or a support service provided by students to the community. This methodology is widely used in university education, specifically in training physical education teacher education (PETE) students. Such programs are planned, coordinated, and integrated into university curricula to optimize learning and meet community needs [1]. SL seeks innovative methodologies to respond to the demands of higher education, reinforces ethical and civic learning among students, and meets higher education institutions’ needs when it comes to interacting with their wider context through social responsibility measures [2,3]. SL represents the union of several key aspects: theory and practice, classroom and reality [4], training and commitment, and cognition and emotion [5,6].

Carson and Raguse [7] state that SL is an ideal strategy for universities and PETE student training to achieve three main objectives: teaching, research, and service provision. SL gives rise to educational experiences that enrich academic study, promote social engagement, and enhance professional and personal skills [8,9]. A number of studies analyze SL for PETE students [10,11,12]. Within the areas of physical education (PE) and physical activity (PA), Carson and Raguse [7] explain that there is a wide range of SL services for PETE students: athletic training programs [13]; recreation [14,15]; health education and promotion [16,17]; rehabilitation and therapy [18,19]; sports management and PE [20].

A number of systematic reviews have focused on SL methodology across different disciplines, such as university social responsibility [21], nursing [22], scientific production [23], and medical education [24]. For instance, a systematic review [7] covered three types of publications: research, overviews of SL programs, and implementation in youth physical activity settings published from 1990 to 2012. Another review [25] on physical education and sports science included publications such as descriptions of educational experiences, research articles, and conceptual papers. To the best of our knowledge, there are no systematic reviews focusing solely on the implementation of SL programs with PETE students.

Research on SL for PETE students has become increasingly prominent as a training resource for students in recent years. Due to its practical nature, there are numerous publications on the use of SL with this group, but only a few have centred around the implementation of PE programs, included scientific data, or been published in peer-reviewed journals.

The results of the implementation of SL with PETE students tend to relate to three main elements: the students themselves, the community, and the SL program [26,27]. However, many existing studies focus solely on analyzing students, overlooking community participants and the SL program itself. Some authors also establish subcategories within the analysis of students [28], who analyzed academic, personal, social, and civic characteristics [6,27,29].

Studies on SL for PETE students continue to be published, but there is no consensus as to their objectives. There is a need to determine whether SL really works and whether the results obtained from these interventions are positive. To this end, this review provides the levels of evidence of a selection of existing studies and analyses the duration of the interventions, the research methodology used, and the focus of the research: students, the community, or the SL program.

The purpose of this systematic review is twofold. Firstly, it aims to analyze the characteristics of studies on the implementation of SL programs with PETE students that have been published in peer-reviewed journals and to identify their objectives: to assess the benefits for students and/or community participants, and/or to evaluate the effectiveness and/or quality of the SL programs themselves. Based on the literature reviewed, our second objective was to propose guidelines to help the scientific community to improve the implementation and quality of SL interventions in PETE.

## 2. Materials and Methods

### 2.1. Search Strategy

The search process was carried out following the protocol outlined in the PRISMA statement. A comprehensive search was conducted in the following databases: Web of Science (WOS), SPORTDiscus (EBSCO), and SCOPUS. Individual searches of all peer-reviewed studies published between 2013 and 2020 were performed. An 8-year window was applied to include only the most recent studies involving the implementation of SL with PETE students. The last search was conducted in November 2020. Search terms synonymous with “service learning” were used in combination with the search terms “physical activity” and “physical education”. Searches were conducted in English and Spanish. Only original articles were included in this study.

### 2.2. Selection Criteria

Potentially relevant studies for this review were checked against the following selection criteria (PRISMA #6) [30]: (a) the study had been published in an international peer-reviewed journal; (b) the study included the implementation of SL with PETE students; (c) the study reported on the implementation of PE, PA, or sports interventions; (d) a full-text version was available in English and/or Spanish. Theses, book chapters, and articles focusing on the discussion of methodological strategies were excluded from this review because their methodological designs lacked empirical rigour. Duplicates were discarded. The study selection process consisted of screening the titles and abstracts identified during the search. Potentially relevant full-text studies were independently checked for eligibility by two researchers. Discrepancies in the selection of the articles were resolved by discussion.

**Figure 1:**
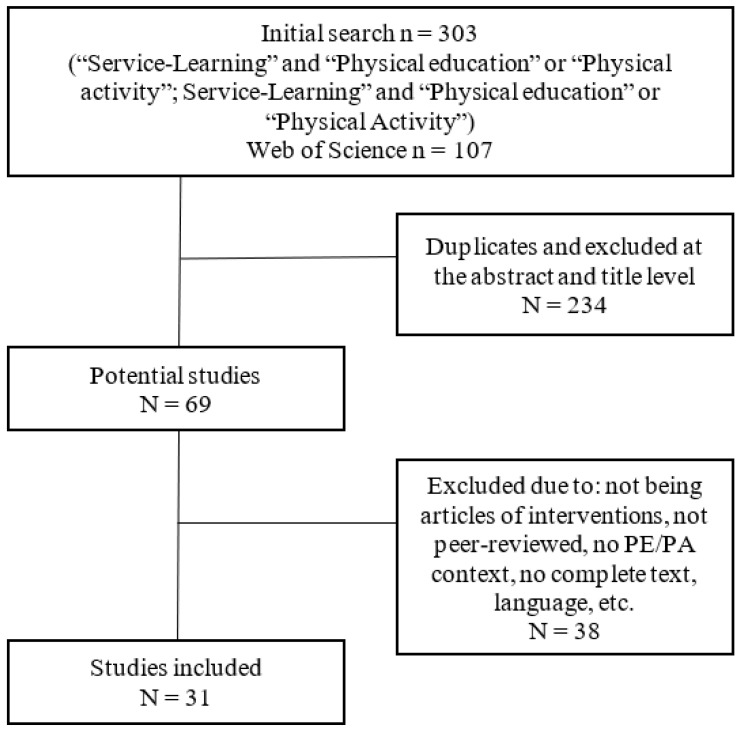
Flow chart of the sampling process.

A flow chart was prepared based on the recommendations listed in the PRISMA statement. A total of 303 studies were retrieved from the literature search. Of these, 234 studies were discarded for failing to meet the inclusion criteria, leaving 38 potentially relevant studies. The full texts of these studies were examined in greater detail. A total of 26 of them failed to meet the inclusion criteria. As a result, a total of 31 articles were included in the systematic review. Figure 1 shows the sampling process used.

### 2.3. Data Extraction and Reliability

Data extraction was carried out independently and consistently by two reviewers (A.N. and R.P-O), who read all the titles and abstracts. Discrepancies were discussed until a consensus was reached. The studies were summarized, and the potentially relevant papers were screened for retrieval. Pilot test forms were used to extract data from the studies. A content analysis of the articles included in this review was also performed. Subsequently, the data were discussed and confirmed by the researchers. The following categories were defined a priori using the method suggested by Harris et al. [31]: authors; journal; year; name of study; location; objectives; sample size; participant profile; duration of study; data sources; methodological analysis; results.

### 2.4. Quality Assessment and Level of Evidence

The criteria for assessing the quality of the studies included were adapted to the Consolidated Standards of Reporting Trials (CONSORT) Statement [32] as used by Pozo, Grao-Cruces, and Pérez-Ordás [33]. The quality assessment criteria were: (a) the SL intervention was implemented with PETE students; (b) the number of participants in the study; (c) the journal in which the article was published is included in the Journal Citation Reports; (d) the duration of implementation; (e) a description of the methodological process was included. Each item was rated from 0 to 2, as shown in Table 1. The overall quality of each study was assessed by adding the number of positive elements together (with the overall score ranging from 0 to 10). Studies with a total score of 7 or higher were considered to be of high quality (HQ); studies with a total score of 4–6 were considered to be of average quality (AQ); studies with a total score lower than 4 were considered to be of low quality (LQ). Quality was assessed by two reviewers independently. Discrepancies in the assessment of the studies were discussed by the two reviewers until a consensus was reached.

The risk of bias is difficult to ascertain in qualitative, social science studies. Version 5.1.0 of the Cochrane handbook emphasizes that, in many situations, it is not practical or possible to blind participants or study staff in the intervention group.

## 3. Results

### 3.1. Duration of the Intervention Programs

The duration of the interventions ranged from 2 weeks [55] to 4 years [48]. In some studies, the exact duration of the intervention was specified, as in Ruiz et al. [54] who indicate that SL intervention was structured in two 40-min weekly sessions and was 10-weeks long.

### 3.2. Methodology and Analysis

The interventions included in this review followed three distinctive methodological approaches: qualitative (21/31), quantitative (2/31) and mixed (9/31). Studies analyzing the results from PETE students used all three types of methodology. Five studies used experimental and control groups: Chiva-Bartoll et al. [40], Chiva-Bartoll et al. [29], and Capella et al. [38] used a mixed methods approach, while Chiva-Bartoll et al. [42] and Willard and Crandall [58] used quantitative methods.

In qualitative studies, both inductive and deductive study designs were found (21). Several data collection procedures, including interviews (10), self-reflection journals (13), reflective reports (5), focus groups (8), literature reviews (1), videos (3), life stories or biographical records (2), observations and field notes (6), and observation sheets (3), were used. Questionnaires intended for trainees were used in the only study adopting quantitative methods [58]. Mixed methods were also employed (9). The instruments used were questionnaires (8), interviews (5), self-reflection journals (4), reflective reports (4), literature reviews (1), life stories or biographical records (2), observations and field notes (1), focus group (1) and test (1). The most frequently used instruments in these designs were questionnaires analyzing the skills acquired by PETE students.

### 3.3. Summary of the Results

The objectives of the different studies focused on: the benefits of SL programs for PETE students; the benefits of SL programs for the community and the effectiveness and quality of the SL programs.

The most common objective in studies on SL for PETE students was to analyze how SL affected PETE students’ training. A total of 30 studies analyzed the influence of SL on PETE students (29/31); however, other studies had a twofold objective: they also analyzed the benefits of SL programs for community participants (4/31) or the quality of the SL program (3/31).

#### 3.3.1. The Benefits of SL Programs for PETE Students (29/31)

The total number of PETE students in the SL interventions was 1872, ranging from 4 [26,36] to 346 [27].

Three types of benefits for PETE students’ skills were identified in the studies: 1. professional skills; 2. social and personal skills and 3. other.

##### Professional Skills (21/31)

The studies focusing on the effects of SL for PETE students analyzed professional skills training (21/31), including generic, academic and professional skills. Lamoneda [49] observed improvements to communication, planning, and organizational skills.

With regards to the pedagogical skill of reflective teaching, improvements in technical content and methodological strategies were also identified [27,36]. Capella et al. [36] indicated that SL improved future training, awareness of the value of practical training, and learning about PETE practice (conflict management, adaptability, feedback, and evaluation).

SL contributed to the learning of participating students in general [48,50,57]. Heo et al. [48] found that students acquired a greater understanding of the subject, an improved ability to analyze problems, and improved skills and classroom material resources for application to real problems. Wilkinson et al. [57] confirmed that participants learned to combine theory and practice and that the project had a positive impact on their professional development as a result of their participation in applied learning, their work in a multidisciplinary environment, and their support of the community.

Capella et al. [38,39] compared the development of teaching competence among PETE students using two intervention methods from the same SL program. Two groups of PETE students exhibited significant differences in their dedication to the SL program in terms of duration and intensity. The authors provided significant evidence of enhanced teaching competence among students with greater dedication to the SL program. In the same vein, Marttinen et al. [11], Galván et al. [46], and Du Toit [44] concluded that the SL program improved students’ pedagogical knowledge and professional skills. Franco-Solà and Figueras [45] assessed teaching skills using the Framework for 21st Century Learning and found that SL helped to improve cognitive, emotional, and social competences among PETE students.

Other studies assessed the acquisition of professional skills from SL with specific populations (17/31) such as cognitively impaired individuals [52,55], children with disabilities special educational needs [27,34,38,39,43,45,59] children with attention deficit hyperactivity disorder [51], others diversity as cultural dimension, low SES schools, disadvantaged population, minority groups [10,11,35,42,47,52] and older adults [54,58]. Surprisingly, Willard and Crandall [58] found no increase in students’ knowledge of PA and attitudes towards PA with older adults. When comparing the SL intervention with the control groups, the main effect was not statistically significant, with both groups showing no significant increase in their knowledge of ageing (λ = 0.979, F(1,24) = 0.531, *p* = 0.473, = 0.021).

Chiva-Bartoll et al. [41] studied children with special educational needs and obtained an inclusive, critical educational experience that allowed them to link theory and practice in a particularly effective way. In turn, Ruiz et al. [54] reported findings related to academic and professional learning when working with older adults.

##### Social (18/31) and Personal Skills (8/31)

Other objectives were to assess the acquisition of social attitudes and skills and personal skills. The studies focusing on these objectives are shown in Table 2.

According to Capella et al. [26], SL promotes the development of social skills and moral values. These authors concluded that SL also promotes the development of students’ critical thinking, reflective capacity, and skills such as conflict management and flexibility. Chiva-Bartoll et al. [40] found that the two experimental groups significantly improved their social skills and attitudes, unlike the control group. Statistically significant differences between the pre-test and the post-test results for the two experimental groups were found: t(40) = 2.9; *p* < 0.05 for experimental group I and t(40) = 5.98; *p* < 0.05 for experimental group II. As expected, no significant differences between the pre-test and the post-test results for the control group were found: t(40) = 1.11; *p* > 0.05. Chiva-Bartoll et al. [29] also found significant differences (t(106) = 2.94; *p* < 0.05) in the overall results of the experimental group in the Effective Personality Test for University Students (in which ‘effective personality’ is understood as a construct whereby personality traits are related to effective behaviours in professional or academic contexts). Heo et al. [48] found that PETE students developed their empathy skills with SL. These students built relationships with older adults (their community participants) and were less likely to hold negative stereotypes towards them. By contrast, Lamoneda [49] analyzed PETE students’ friendliness, involvement, teamwork, listening skills, and support during the SL program. According to Webster et al. [56], SL can offer opportunities for PETE students to develop knowledge, skills, and attitudes useful for leadership roles. The results of Wilkinson et al. [57] suggest that SL is a contemporary caring pedagogy that prepares future teachers for the realities and challenges of a changing world. Willard and Crandall [58] assumed that contact with older adults would result in more positive student attitudes and greater knowledge of ageing, but failed to find positive results in this case. Finally, Martínez et al. [51] analyzed the influence of SL on students’ social participation and highlighted the importance of the latter among the impacts of the SL methodology.

Chiva-Bartoll et al. [41,42] studied values, personal attitudes, and/or personal life plans, concluding that SL promotes subjective happiness and pro-social attitudes [42]. Ruiz et al. [54] analyzed social sensitivity and disconfirmation of negative stereotypes, satisfaction and personal growth, and desire for social justice, resulting in relevant items for PETE students. Capella et al. [37] reported that SL promoted social entrepreneurship skills in PETE, which represents a highly valuable, innovative educational experience on a personal and social level. Bruce [35] pointed out that SL helps students to become more open and ethically responsible towards others.

##### Other (8/31)

Two studies analyzed changes in PETE students’ identities [27,51]. According to their results, SL is a source of positive feelings that prompt change in students and their values, one of the most prominent of which is empathy.

Martínez et al. [51], Peralta et al. [53], and Lleixà and Ríos [50] studied the acquisition of cultural training and understanding (3/31). Martínez et al. [51] concluded that SL also helped students to overcome stereotypes, contextualize their training, learn to approach PE as a resource for social intervention, and improve their communication and decision-making skills. Peralta et al. [53] reported that PETE students’ perception of their cultural competence had also improved. Differences in pre-service teachers’ perceptions of their cultural competence had improved from baseline (*M* = 59.59; SD = 8.25) to follow-up (*M* = 69.89; SD = 8.70) and were statistically significant (t(54) = −6.81; *p* < 0.001). Gil-Gómez et al. [27] found that PETE students showed a limited understanding of cultural competence. In turn, Lleixà and Ríos [50] viewed the experience as highly positive, as it exposed students to a reality that is often socially stigmatized and difficult to access.

Ruiz et al. [54] analyzed social sensitivity and disconfirmation of negative stereotypes, satisfaction and personal growth, and desire for social justice. In turn, Capella et al. [37] assessed social entrepreneurship skills, while Giles et al. [47] studied emotions in PETE students. In this case, positive views and emotions prevailed among students. The joy of feeling valued and loved by children, as well as welcomed and respected by teachers, was particularly emphasized. Negative emotions became positive, which helped students to build their professional identity through reflection and self-criticism.

#### 3.3.2. The Benefits of SL Programs for the Community

Only four studies focused on community participants, but their objective was twofold: to analyze the benefits of SL programs for PETE students and the community [46,50,54,56]. These studies analyzed elementary and middle school participants, older adults, youth, staff, and parents from a school and inmates.

There are no studies exclusively assessing members of the community. The number of participants in the SL programs studied here could not be specified because most of the studies did not report or study them, or because they varied depending on the day or session [50,56,60].

There were different types of participants: people with special educational needs (15/31); people with other special characteristics (victims of disasters, individuals of low socioeconomic status, specific ethnic groups) (8/31); primary and secondary school children (3/31); the school community as a whole (staff, students, and parents) (1/31); elderly people (3/31); prisoners (1/31); and others (1/31).

Regarding the implementation of an SL program at a school, Webster et al. [56] concluded that SL was a viable strategy to increase opportunities for promoting PA among children, staff, and parents. The benefits of SL for prisoners in Lleixà and Ríos [50] related primarily to the impact of PA and sport on their socialization, communication, and personal skills. This study found that SL provided prisoners with the sense of optimism needed to overcome their deprivation of liberty while also keeping them in touch with reality, helping them to release tension, increasing their expressive abilities, giving them a break from their routines, and other benefits related to hygiene and motor and physical improvements. Ruiz et al. [54] identified four emerging categories among the benefits of SL with older adults: disconfirmation of negative stereotypes, improvement of physical function, satisfaction and desire of continuity, and social interaction.

#### 3.3.3. The Effectiveness and Quality of the SL Programs

Three studies analyzed the effectiveness of SL programs [10,49,52]. Lamoneda [49] focused on whether a sports recreation program during school break times was providing a good quality service. The following elements were assessed: teachers, facilities, activities, and relationships with staff. In the overall assessment, the sports promotion services were considered to be acceptable (3.42 ± 0.5). Regarding the sports initiation program, there was room for improvement with regard to its duration (2.69 ± 0.9) and the number of sessions per week (2.44 ± 1.1). In the sports entertainment program, satisfactory results were obtained for all items. The overall evaluation of the sports promotion services suggested that the activity was good (4.11 ± 0.6). The aspects to be improved in the sports entertainment program included updating the activities (3.67 ± 0.7) and increasing the number of sessions (3.78 ± 0.8). MacPhail and Sohun [10] studied a course-embedded SL project in a physical education teacher education program to provide a broader, potentially more critical view of the experience, knowledge, and learning related to our effort to link service and learning. They concluded that linking academic coursework with community service structured through reflective practice is a challenge, and that there is a need to invest more time so that PETE teachers and students engage in dialogue with one another focusing specifically on SL. Santos et al. [52] addressed the limitations of SL in the training of PETE students. They concluded the following: there is a lack of training in project design, implementation, and evaluation; SL places a heavy workload on PETE students and teachers; it is difficult to coordinate everyone involved.

Table 3 provides an overview of the data obtained from each of the 31 empirical articles reviewed: authors, objectives, number of participants, program recipients, instruments used, research methodology, and main results.

## 4. Discussion

The purpose of this systematic review was twofold: to analyze the characteristics of studies on the implementation of SL programs with PETE students and, in view of the shortcomings identified, to propose future lines of research on SL in PETE. The results from the first study objective can be grouped into three categories: the benefits of SL programs for PETE students, the benefits of SL programs for the community, the effectiveness and quality of the SL programs. In this review, most studies (29 studies) focused on students, while four focused on community participants. Only three studies examined the effectiveness and quality of services provided in the SL program. These data are consistent with research in other areas, where the community is outside the scope of the study [7,80,81]. In addition, results focusing on PETE students were classified into three types of benefits or skills: professional skills; social and personal skills; and other skills (identity, vocational skills, cultural competence, emotions, etc.). This classification is very similar to that used by other authors [51], who refer to skills as competencies. This review found that 28 of the 31 selected studies reported positive results in the acquisition of professional, social, and personal skills by PETE students. These data are in line with the conclusions of meta-analyses of SL programs in other fields (nursing, medicine, social work) with participants at different educational levels (primary, secondary, and higher education, and vocational training) [82,83,84]. Celio et al. [82] conducted a meta-analysis of 62 studies with 11,837 students who participated in SL interventions and made significant progress in five outcome areas: personal and academic skills, attitudes towards school and learning, civic engagement, and social engagement, all of which are consistent with the findings identified in this study. SL helped to promote the development of social, moral, and personal skills, and the findings in this review coincide with those of Yorio and Ye [84]. Their review of 40 studies involving business and management scholars reported the effects of SL on understanding social issues, social awareness and sensitivity, perception of disabled individuals, interpersonal skills, ethical and moral values, responsibility, community engagement, and personal insight. The findings in this review are also consistent with Puig [85], who views SL as a methodology promoting improved social relationships. This improvement occurs in the following ways: through collaboration between colleagues in community service tasks, by improving relationships between participants, by contributing to the common good, and through citizen participation. SL enables a cultural shift towards values such as solidarity, social cohesion, equality, environmental engagement, and social responsibility. Only one of the studies obtained unexpected results concerning the acquisition of knowledge and attitudes towards PA with older adults [58]. Interestingly, this was one of the two studies using a quantitative method. This raises the question of whether an exclusively quantitative approach is appropriate for this type of research.

In this review, only four studies analyzed the benefits of SL programs for the community. SL interventions proved to be positive for the community subjects, and their objectives were achieved in all the cases studied. These data are consistent with those reported by Doolittle and Rukavina [86] and Jones et al. [87]. It is clear that SL would be meaningless without the contribution that it makes to the community [88]. Therefore, studies should analyze whether the objectives for the community are met and whether the methods used are appropriate to benefit community participants. Sallis [89] adds that, in order to achieve real, tangible, long-lasting benefits, multi-level work strategies must be prioritized and studies must include an analysis of the benefits for the community. Chiva et al. [90] argue that evaluation of the social impact of SL has been neglected and propose a model for evaluating the impact of SL in PETE, with special emphasis on its social dimension.

In this review, only three authors [10,49,52] analyzed the effectiveness of the SL program and focused on assessing the quality of the service provided. Lamoneda [49], assessed: teachers, facilities, activities, and relationships with staff. According to the results, the duration of the program and the number of sessions needed to be improved, corroborating the findings of Conway et al. [83], who examined the impact of specific elements of the program (moderators) on the degree of change seen in participants. Other researchers also emphasize that the duration, structured reflections, and number of service hours offered by SL programs should be improved [12]. In this vein, Eyler et al. [91] and Tannenbaum and Berrett [92] list several sources demonstrating the benefits of increasing the intensity and duration of SL programs [93]. In turn, Santos et al. [52] have designed and validated a scale for evaluating SL programs with PETE students to standardize their evaluation and validate their quality and effectiveness.

Regarding the second objective of this research, analysis of the results of the studies has allowed us to identify several potential lines of work to ensure that SL programs in PETE produce better outcomes for all stakeholders, which may become possible lines of research. It is essential to analyze all the actors involved in SL programs in PETE. There is a lack of research assessing community participants, which is consistent with studies [94], who authored an article focusing exclusively on this shortcoming in SL programs. It seems contradictory to implement programs with the aim of helping the community and neglect to evaluate whether these objectives are actually attained. The effectiveness of SL is only relevant if the community objectives are met [10,91], requiring examination of these objectives in order to confirm this effectiveness. Lleixà and Ríos [50] concluded that their experience demonstrated that interactive dialogue between the different stakeholders in SL could actively promote collaborative learning. Blouin and Perry [95] noted that there is a wealth of research reporting numerous pedagogical and personal benefits for students, such as improved grades, greater civic engagement, and increased understanding and appreciation of diversity, but there are few studies on the impact of SL on the community.

The quality of the program was only analyzed by three of the studies. We believe that focusing on program quality would benefit both students and community participants and that further research is required in this area. The quality of SL programs for PETE students should be assessed as a determinant of their effectiveness. In addition, Blouin and Perry [95] discuss obstacles to effective SL: problems related to student behaviour, lack of communication between instructors, and problems in the organizations themselves. These quality-related factors should also be considered and studied.

We observed significant heterogeneity in terms of the research methods and techniques employed, as well as disadvantages in the use of exclusively quantitative methods. For this reason, we believe that mixed methods are more appropriate. These data are consistent with those observed in other systematic reviews [51], whose research focused on SL and its interaction with university social responsibility. Only one in 24 studies used a quantitative method [96]. Willard and Crandall [58] argue that future researchers should consider the collection of qualitative data in the form of student reflections, as they are already a crucial tool in the study of SL.

The limitations of this study are rather similar to those found in reviews of SL in other fields, such as nursing, medicine, and social work. It is possible that only papers with positive results were published. The strength of this study lies in its specific analysis of the implementation of SL programs with PETE students, which had not yet been studied. With regard to future lines of research, we propose the following: using mixed methods, coordinating the different stakeholders, and studying community participants and the quality of the programs themselves as well as students.

## 5. Conclusions

This article sought to analyze the benefits of SL programs for PETE students and it is safe to say that, as a learning methodology, SL has excellent potential as a resource for developing professional, personal, and social skills in PETE students. SL also promotes participation in teaching by connecting future PE professionals to the realities and challenges of a diverse and constantly evolving educational environment. This produces benefits for the community and connects education to the real world. Teaching, research, and service objectives can be accomplished by including all stakeholders in the SL process. With respect to the guidelines to help the scientific community to improve the implementation and quality of SL interventions in PETE, we believe that further studies are needed to analyze all three factors: PETE students, community participants, and program quality. There is a need for a mixed research methodology that compiles contributions from all stakeholders. The effectiveness of the studies should also be assessed via longer implementation periods, as this could result in more objective, controlled measurements and more generalizable findings.

## Figures and Tables

**Table 1:** List of studies included and quality level.

Study	Description of the Program	Number of Participants	Included in JCR/SJR	Duration of the Program	Description of the Methodology	Overall Score	Quality Level
An [34]	2	0 (*n* = 10)	1	2	1	6	AQ
Bruce [35]	0	2 (*n* = 32)	1	0	1	4	LQ
Capella et al. [36]	1	0 (*n* = 4)	1	2	2	6	AQ
Capella et al. [26]	0	0 (*n* = 4)	1	2	2	5	AQ
Capella et al. [37]	1	2 (*n* = 32)	1	2	2	8	HQ
Capella et al. [38]	1	2 (*n* = 96)	1	2	2	8	HQ
Capella et al. [39]	1	2 (*n* = 96)	1	2	2	8	HQ
Chiva-Bartoll et al. [40]	1	2 (*n* = 108)	2	2	2	9	HQ
Chiva-Bartoll et al. [29]	1	2 (*n* = 149)	1	2	2	8	HQ
Chiva-Bartoll et al. [41]	1	2 (*n* = 169)	1	0	1	5	AQ
Chiva-Bartoll et al. [42]	1	2 (*n* = 104)	1	0	2	6	AQ
Douglas et al. [43]	2	0 (*n* = 10)	0	2	2	6	AQ
Du Toit [44]	1	2 (*n* = 140)	1	2	1	7	HQ
Franco-Solà and Figueras [45]	2	0 (*n* = unknown)	1	0	2	5	AQ
Galvan et al. [46]	1	1 (*n* = 16)	2	0	2	6	AQ
Gil-Gómez et al. [27]	0	2 (*n* = 346)	2	2	2	8	HQ
Giles et al. [47]	0	2 (*n* = 42)	1	2	2	7	HQ
Heo et al. [48]	2	2 (*n* = 142)	1	2	2	8	HQ
Lamoneda [49]	2	2 (*n* = 50)	0	2	2	8	HQ
Lleixà and Ríos [50]	1	0 (*n* = 10)	0	2	2	5	AQ
MacPhail and Sohun [10]	2	2 (*n* = 68)	2	2	1	9	HQ
Martínez et al. [51]	1	2 (*n* = 25)	1	2	2	8	HQ
Marttinen et al. [11]	2	0 (*n* = 9)	2	2	1	7	HQ
Santos et al. [52]	1	2 (*n* = 32)	1	2	1	7	HQ
Peralta et al. [53]	2	2 (*n* = 55)	1	2	2	9	HQ
Ruiz et al. [54]	1	2 (*n* =23)	2	0	2	7	HQ
Ward et al. [55]	2	0 (*n* = 8)	2	0	2	6	AQ
Webster et al. [56]	2	1 (*n* = 18)	2	2	2	9	HQ
Wilkinson et al. [57]	1	0 (*n* = 6)	2	2	2	7	HQ
Willard and Crandall [58]	2	2 (*n* = 27)	0	0	2	6	AQ
Woodruff and Sinelnikov [59]	0	2 (*n* =50)	2	0	2	6	AQ

Parameter 1: did the study provide a detailed description of the SL implementation program? 0: not included; 1: description included, but it is short and imprecise; 2: detailed description included. Parameter 2: number of participants: 0: from 1–10 participants; 1: from 11 to 20 participants; 2: more than 20 participants. Parameter 3: the article is included in JCR (Journal Citation Research): 0: not included; 1: included in Scimago Journal Rank (SJR); 2: included in JCR. Parameter 4: duration of the intervention: 0: less than 3 months; 2: more than 4 months. Parameter 5: did the study inform about the methodological process applied? 1: informed but incomplete; 2: informed.

**Table 2:** Objectives of the studies.

	PETE				COMMUNITY			SL PROGRAMME
	1	2	3	4	5	6	7	8
An [34]	x		x					
Bruce [35]			x					
Capella et al. [36]		x	x					
Capella et al. [26]	x							
Capella et al. [37]			x	x				
Capella et al. [38]	x							
Capella et al. [39]	x							
Chiva-Bartoll et al. [40]		x						
Chiva-Bartoll et al. [29]			x					
Chiva-Bartoll et al. [41]	x	x	x	x				
Chiva-Bartoll et al. [42]	x	x						
Douglas et al. [43]	x		x					
Du Toit [44]	x	x	x					
Franco-Solà and Figueras [45]	x	x						
Galvan et al. [46]	x				x			
Gil-Gómez et al. [27]	x			x				
Giles et al. [47]				x				
Heo et al. [48]		x	x					
Lamoneda [49]	x	x	x					x
Lleixà and Ríos [50]				x		x	x	
MacPhail and Sohun [10]								x
Martínez et al. [51]	x		x	x				
Marttinen et al. [11]	x		x					
Santos et al. [52]								x
Peralta et al. [53]	x			x				
Ruiz-Montero et al. [54]	x		x	x	x	x	x	
Ward et al. [55]	x		x					
Webster et al. [56]	x		x		x			
Wilkinson et al. [57]	x		x					
Willard and Crandall [58]	x		x					
Woodruff and Sinelnikov [59]	x		x					

Note: PETE: 1. Professional skills; 2. Personal skills; 3. Social skills; 4. Others; COMMUNITY: 5. Physical skills; 6. Social skills; 7. Personal skills; SL PROGRAMME: 8. Effectiveness and quality.

**Table 3:** General overview of the literature review.

Authors	Objectives	(Pete) *N* =	Program Recipients	Instrument	Methodology	Results
An [34]	To explore the influences of SL on the understanding of PETE of disability and their learning of how to teach students with disabilities (SWDs).	10	22 children with disabilities	Semi-structured and face-to-face interviews, reflective journals, visual artefacts, and field notes.	Qualitative: case study	Three themes emerged from the thematic analysis: challenging but fulfilling experiences, uncovering the qualities and roles of teachers, and transforming perceptions of disability and teaching. Participants perceived the program as supporting their learning of disability and the teaching of SWDs because it enabled them to learn in real-life settings.
Bruce [35]	To trial a post-critical approach to SL within a PETE context and to consider the extent to which this approach may invite PETES into a radically different encounter with the Other	32	Community contexts radically different cultural	Journal reflections of investigator, and PETE student journals.	Qualitative: through employingMaxwell and Miller [61] categorizing and connecting the data analysis method, it was coded, compared and then generated themes from investigator and student’s journal entries.	Rather than repressing uncertainty and trauma, this SL project was in essence an invitation to experience trauma, violence and difficult situations, in order that PETE students as future teachers may consider something of what it means to be in a position of openness, and ethical responsibility towards the Other.
Capella et al. [36]	(1) To reveal participating students’ personal traits. (2) To assess whether SL can be used to develop social skills and moral values. (3) To assess the suitability of life stories as a tool in SL educational research.	4	Children with functional diversity	Open-ended interviews with structured questions.	Qualitative: biographical methods within biographical records [62].	Social skills and values were acquired. The suitability of life stories as a research tool in this field was verified.
Capella et al. [26]	(1) To verify whether SL promotes students’ critical thinking skills. (2) To ascertain whether SL is useful for developing practical skills and PE content.	4	Children with functional diversity	Open-ended interviews with chronological narration of experiences.	Qualitative: life stories.	SL improved future training, awareness of the value of practical training, and learning about PETE practice (conflict management, adaptability, feedback, and evaluation).
Capella et al. [37]	To examine the effects of a SL project on Social Entrepreneurship Competency (SEC) in PETE students.	32	Coaching Corps	Quantitative: Social Entrepreneurship Competency Scale [63] Qualitative: semi-structured and face-to-face interviews, reflective journals, visual artefacts, and field notes.	Mixed methods study that uses methodological triangulation.	SL promoted SEC in the PE field, representing an educational experience of great value on a personal, social and innovative level. The connection between the different personal, social and innovative aspects that make up the SEC is highlighted, pointing out that they were developed jointly and reciprocally. In addition, it is appreciated that the SL program caused a very similar impact on the members of the experimental group, thus pointing to the homogeneity of its effect.
Capella et al. [38]	To compare the development of teaching competency in pre-service teachers of PE through two different modalities of intervention from the same SL program.	96	Children with motor-functional diversity (n = 150)	Mixed methods with methodological triangulation: the TC/MSBLG-R instrument [64] and life histories.	Mixed: Quantitative: quasi-experimental design of two non-equivalent experimental groups implementing the TC/MSBLG-R instrument [64]. Qualitative analysis: by elaborating life histories of multiple crossed stories. Two groups of PETEs: there were important differences in their dedication in terms of duration and intensity. Group 1: 30 sessions, Group 2: just 9 sessions.	Quantitative results provide significant evidence regarding the academic effect of SL on pre-service teachers while qualitative interpretation complements this view, reflecting how this learning was developed.
Capella et al. [39]	To compare the development of teaching competence through two modalities of intervention from the same SL program.	96	Children with motor-functional diversity (n = 150)	Mixed methods with methodological triangulation: rubric that measures teaching competence when applying motor and expressive games (CDJME) [26] and life histories.	Mixed: pretest-postest and postest-postest. Tests: Cronbach’s alpha, Kolmogorov-Smirnov test, Mann-Whitney *U*-test, Wilcoxon’s signed-rank test, and Spearman’s rho. A qualitative analysis was performed on 12 of the interviews using several life histories of multiple crossed stories. Two types of sampling methods were used to select these interviews: quota sampling (four) and chain-referral sampling (eight).	The quantitative results provide significant evidences regarding the promotion of teaching competence among students (*p* < 0.01), the qualitative interpretation complements this view explaining how this competence was developed. In addition, the data transformation highlights a remarkable presence of each aspect analyzed in the discourse of the interviewees. Finally, we conclude that the implementation of the SL program enhanced teaching competence of university students, at the same time as additional academic learnings were promoted.
Chiva-Bartoll et al. [40]	To analyze the effects of SL on the development of ‘Effective Personality’ in the training of PE students.	108	Children with functional diversity	Effective Personality Test for University Students [65]. Semi-structured interviews. Follow-up journals.	Mixed: interviews and follow-up journals with a multi-phase approach, open coding, and axial coding. Post-test using the Effective Personality Test for University Students [65].	The Effective Personality Test for University Students showed significant differences in favour of the experimental group.
Chiva-Bartoll et al. [29]	To improve an SL program to promote the acquisition of three categories of social skills and attitudes: group awareness, engagement and group organization skills, and communication skills.	149	Children with physical disabilities	Questionnaire [66].	Mixed: Qualitative: interviews and follow-up journals. Quantitative: quasi-experimental design with three non-equivalent groups. Questionnaire [66].	The quantitative study showed that, unlike the control group, both experimental groups improved their social skills and attitudes after the SL program.
Chiva-Bartoll et al. [41]	To analyze: the impact of the program SL had in PETE from an inclusive perspective (technical dimension); learnings about diversity of the children (cultural dimension); interpretation of the social distribution of power (political dimension); their scales of values, personal attitudes, and/or personal life plans (post-structural dimension).	169	116 children with special educational needs	Reflective journals	Qualitative: reflective journals were used as an instrument to gather information from their experiences.	SL had a positive impact on PETEs’ training, helping them to have an inclusive and critical educational experience that allowed them to link theory and practice in a truly operative way. They support the adequacy of proposing critical perspectives both in the research and in the application of SL programs.
Chiva-Bartoll et al. [42]	To analyze the effects of a SL program on the subjective happiness (SH), prosocial behaviour (PB), and professional learning (PL) perceptions of PETE students.	104	Disadvantaged population	Subjective Happiness Scale, the Prosocial and Civic Competence questionnaire, and the Impact of SL during Initial Training of PA and Sports questionnaire (IMAPS-AFD-FI is a validated tool to analyze SL experiences in the context of PE [67].	Quantitative: quasi-experimental design of two non-equivalent groups (experimental and control) with pre-test and post-test measures to compare how the participation in a SL program based on PA promotion affected the SH, PB, and PL of PETE students. In addition, to deepen on the analysis, the correlations among these variables were also analyzed.	SL only had a significant influence on SH when the students compared themselves with their peers. The effect of SL on promoting PB and PL perceived was significant in several of their dimensions and there are a correlation of the perceived PL with the PB than with the SH.
Douglas et al. [43]	To explore the meaning that disability-related simulations had on preservice teachers’ perceptions of individuals with disabilities and to what extent these experiences changed their beliefs and values about teaching students with disabilities in PE.	10	People with disability and diversity	Reflective participant narratives and a semi-structured focus-group interview	Qualitative: data from reflective participant narratives and a semi-structured focus-group interview were collected, transcribed and thematically analyzed to reveal five themes: perceived treatment, mobility challenges, meta-perceptions, changes in perceptions of impairment, and future impact on teaching students with disabilities.	The findings present profiles of preservice teachers’ perceptions of disability and learning outcomes, and highlight the potential impact disability- related simulations can have for preservice teachers to gain empathy for impairment, and the resulting development of more thoughtful approaches to teaching students with disabilities in PE.
Du Toit [44]	To investigate the benefits and challenges experienced by pre-service and in-service teachers in a SL (PETE), in a South African school setting of unqualified PE teachers and a lack of PE equipment.	140	Pre-school and primary school in a low- to middle socio-economic area	Reflections, interviews and questionnaires	Mixed: qualitative data were analyzed using an interpretive approach, while quantitative data were analyzed using descriptive statistics.	The results show that pre-service and in-service teachers perceived the program as beneficial to all role players. Unique contributions of this study lie in the experiences of the teachers that the barriers of teacher incompetence and a lack of PE equipment were overcome due to the SL program.
Franco-Solà and Figueras [45]	To assess teaching skills using the Framework for 21st Century Learning. To collect evidence of the effectiveness of SL in the acquisition of competences linked to specific learning and innovation skills, as well as personal and professional development skills.	unknown	Hospital Guttmann: children with low mobility and functional diversity	Rubrics, reflective journals and videos	Qualitative: rubrics with Likert scales, journals and videos.	Applying learning content in real situations makes SL and PE a pedagogically consistent and reciprocally useful dyad. University teaching experiments demonstrate the potential of SL methodology, which activates student learning in all its dimensions: cognitive, emotional, and social. Acquiring knowledge through real-life practice transforms knowledge into competence.
Galvan et al. [46]	To answer: (a) what benefits, if any, did the children and adolescents gain from participating in the SL program? and (b) Did the integration of teaching models in a SL course enhance the knowledge base for teaching among preservice educators?	16	Elementary and middle school participants n = 50	FITNESSGRAM one-mile run test and journal reflections, and focus group interviews.	Mixed-methods: Quantitative: (participants’) data consisted of a pretest–posttest design to determine the effect of a fitness training program on cardiorespiratory endurance. FITNESSGRAM one-mile run test to measure cardiorespiratory endurance. Qualitative: (PETE students) qualitative data preservice teacher’s journal reflections, two focus group interviews.	Findings revealed a significant improvement in cardiorespiratory endurance among students, while qualitative data provide evidence of increases in general pedagogical content, knowledge of curriculum, and knowledge of educational contexts among teachers.
Gil-Gómez et al. [27]	To analyze the contribution of SL: (1) To technical content and methodological strategies. (2) To technical knowledge that SL offers pre-service teachers with respect to teaching children with special educational needs. (3) How SL contributes to their cultural understanding of diversity. (4) How SL produces changes in pre-service teachers’ identities.	346	Children with special educational needs or limited motor development	Individual diaries, focus groups.	Qualitative: Butin’s model structure [68]. Data coding software and groups of experts were used.	SL allowed pre-service teachers to acquire skills that improved teaching competency, especially when working with children with SEN. SL increased cultural understanding of disability, had an impact on the identity of pre-service teachers, and led to changes in their conception of socio-cultural reality, especially in understanding disability.
Giles et al. [47]	To analyze the emotions experienced by undergraduate students enrolled in the SL program.	42	Schools in contexts of social exclusion	Reflective journals from the identification of the critical incidents experienced throughout it [69,70].	Qualitative: analysis model in a categorical approach to the emotions narrated by the participants.	Positive views and emotions prevailed among students. The joy of feeling valued and loved by children, as well as welcomed and respected by teachers, was particularly emphasized. Negative emotions became positive, which helped students to build their professional identity through reflection and self-criticism. However, the fear of failure and of being unable to rise to the occasion remains latent and can sometimes cause anxiety.
Heo et al. [48]	To explore the learning outcomes of undergraduate students who facilitated a sporting event for older adults.	142	Individuals aged 50 and older	Reflective essays.	Qualitative: reflective essays and content analysis including comparisons, contrasts, and categorizations [71].	The students developed relationships with older adults, were less likely to negatively stereotype them, and realized the importance of maintaining an active lifestyle. Self-esteem and a sense of social responsibility may have also been increased.
Lamoneda [49]	(1) To assess whether SL contributed positively to PETE students’ academic learning and general skills. (2) To explore whether sports recreation programs during break times offered good quality services.	50	Primary and secondary education students	Questionnaire about quality in PA programs (ICPAF) [72]. General skills: ad hoc questionnaire.	Mixed: qualitative analysis and a post-intervention assessment (self-assessment, teacher training report, and an analysis of the activity’s contributions to competence development). Quantitative analysis: the ICPAF questionnaire [72] and an ad hoc questionnaire for measuring students’ general skills.	SL was considered to be suitable for this purpose because of its contributions to instructor training and general skills. Limitations in service quality were identified.
Lleixà and Ríos [50]	(1) To determine the impact of SL on inmates; (2) to evaluate student learning.	10	Inmates (*N* = 8)	Individual diaries, focus groups sessions, semi-structured interviews.	Qualitative: focus groups (11 participants and 8 inmates) and field diaries. Two focus groups; one semi-structured interview with a representative of the prison officers; and students’ field diaries.	The impact of PA on inmates’ socialization was demonstrated by improvements to communication and personal skills. The students gained knowledge, relating especially to the contextualization of learning.
MacPhail and Sohun [10]	To interrogate a course-embedded SL project in PETE	68	Sport partnership organization (Target groups included young people, older adults, minority sports groups, disability groups, disadvantaged communities, sports clubs, walking groups, teenage girls, schools and the unemployed)	Interviews, focus groups, short narrative responses and course-specific survey.	Qualitative: due to the qualitative nature of the data, interviews, focus groups, narrative responses and open-ended questions from the course-specific survey were analyzed using thematic content analysis. Approaching the study inductively, the authors considered the data in detail using an ‘open’ coding system to develop the initial categories [73].	The coding of the data provided rich evidence on the extent to which the relationship among three main elements of SL that is, academic coursework, community service and reflective practice [74], resulted in a meaningful, relevant and worthwhile SL experience for the PETE. Meaningful interaction among the three is necessary for the effective fulfilment of each and this is strongly conveyed in the results that follow under the headings of: (a) relevance (or not) of the course outcomes to the different stakeholders; (b) SL activity in the community; and (c) delivery of the course.
Martínez et al. [51]	(1) To analyze the technical knowledge of ADHD offered by SL. (2) To assess the contributions made by SL to cultural understanding of diversity. (3) To analyze the influence of SL on students’ social participation. (4) To assess changes in students’ identities after the program.	25	Children with attention-deficit hyperactivity disorder	Focus group diaries.	Qualitative: focus group sessions following Butin’s model of categorical content analysis [68]. Written reflections [75] and focus group sessions.	Technical, cultural, and identity-related aspects improved, but there were limitations in terms of social participation. Teaching skills, academic skills, self-efficacy, and problem-solving skills improved. There was a greater understanding of the abilities, interests, and needs of children with ADHD.
Marttinen et al. [11]	To understand the experiences of PETE students a SL program. The research questions that guided this research were: (1) how does an afterschool SL program that utilizes pre-service teachers develop students’ pedagogy? (2) What are the experiences of pre-service teachers teaching in low SES schools through a SL approach?	9	Students at a low SES school.	A total of 11 semi-structured interviews [76] and weekly journal that was completed on Google Docs where each PST was given access to contribute to a running document to give their reflection of the week	Qualitative: case study.	Three themes emerged from the data analysis. Theme 1—developing pedagogies in real-world settings. Theme 2—connecting with students and learning how to manage behavior. Theme 3—teaching in a low SES school: a wake-up call. Pre-service teachers in this study were able to practice their pedagogy in a real-world environment and gain valuable experience in developing classroom and behaviour management skills. This program provides a model for a SL approach where pre-service students can practice and refine their teaching skills through extended involvement in an after-school program that served students in a low SES community.
Santos et al. [52]	To know and analyze the limitations that SL presents in PETES training in the university context	PETES 30 TEACHERS 2	University students with intellectual disabilities (n = 61)	Teacher journal, group interviews and journals PETE students.	Qualitative: Teachers. Teacher’s journal: this is an open-ended instrument for collecting information on the SL experience (initial stage, diagnosis, intervention, and evaluation) and assessing the strengths and weaknesses detected, as well as providing recommendations for improvement. Students. Group interviews in which all members of the working group participated. These were conducted at the end of the project to assess the strengths and weaknesses of the project and the learning process. A portfolio (individual and group journal) compiling the different tasks students carried out, as well as an individual and group journal with students’ experiences analyzing the difficulties encountered and their potential solutions.	Three important points are highlighted: (1) the lack of training and experience of university students in the design, implementation, and evaluation of sports and physical activity projects; (2) the high workload for both teachers and students; and (3) the difficulty of coordinating programs, teachers and students, and students themselves.
Peralta et al. [53]	(1) To explore students’ expectations prior to an SL program with the Aboriginal community. (2) To assess pre-service teachers’ cultural knowledge, skills, and teaching abilities.	55	Native community	Individual interviews, group interviews, literature reviews, journals, the Multicultural Teaching Competency Scale [77].	Mixed: quantitative data. The Multicultural Teaching Competency Scale [77]. Qualitative data from formal interviews, individual and group reflections, and focus group interviews.	Students’ perceptions of their own cultural competence, knowledge, skills, and attitudes improved.
Ruiz et al. [54]	To analyze the effects of an intergenerational SL program from the complementary perspective of the different agents involved (students and older adults).	23	20 older adults	Reflective journals were used for PETE students and semi-structured group interviews for older adults.	Qualitative: PETE: journals followed a semi-structured scheme, in which there were open questions to expose general perceptions and closed ones concerning more specific learnings. Older adults: three semi-structured group interviews whit open questions	The following categories emerged from PETE students: social sensitivity and disconfirmation of negative stereotypes, academic and professional learnings, satisfaction and personal growth, and desire for social justice. From older adults, four complementary categories emerged: disconfirmation of negative stereotypes, improvement of physical function, satisfaction and desire of continuity, and social interaction.
Ward et al. [55]	To explore PETE students experiences of cognitive disequilibrium theory during a SL project.	8	Children from the Pacific islands with a low-middle socioeconomic status	Formal interviews, videos of planning, videos of teaching, videos of reflection sessions.	Qualitative: formal interviews supported by secondary data sources including videos of planning, videos of teaching, videos of reflection sessions, and informal interviews.	The SL programs had potential for training PE teachers, guiding them in their professional work, and providing opportunities for them to teach in diverse, authentic situations.
Webster et al. [56]	(1) To examine PETE students SL experiences of planning and implementing comprehensive PA course assignments at a school. (2) To promote PA before, during, and after school for youth, staff, and parents.	18	Youth, staff, and parents from a school	Interviews, observation sheets, contributions from teachers.	Qualitative: focus group interviews, written reflections, field notes, and artifacts. Constant comparison techniques and triangulation. Inductive analysis, grouping of concepts obtained from the data, and open and axial coding methods.	This study provided insight into the feasibility of the SL program with PE students and revealed promising aspects and potential problems regarding its implementation.
Wilkinson [57]	To explore the experiences of PETE students in a SL project for children with ADHD.	6	Children with attention-deficit hyperactivity disorder	Videotaped individual semi-structured interviews. Participants’ unit plans, lesson plans, and written reflections.	Qualitative: phenomenological reduction, data coding, and subject identification [78].	The SL project motivated students to devote their careers to teaching. Students learned how to merge teaching theory and methods in practice. Working in a multidisciplinary manner and helping the community had an impact on students.
Willard and Crandall [58]	(1) To examine the effects of SL on PE students. (2) To assess whether contact with older adults would result in more positive attitudes towards this population.	27	Older people	Demographic questionnaire, Palmore’s Facts on Aging Quiz (PAQ) [79], Fraboni Scale of Ageism (FSA).	Quantitative: t-tests and Pearson chi-squared tests. Comparisons between the experimental group and the control groups were made using analysis of variance.	No significant differences between the experimental group and the control groups were found. It was concluded that it would be more enriching to conduct mixed methods research in which qualitative data were also recorded.
Woodruff and Sinelnikov [59]	To examine what the students learning to teach young adults with disabilities consider meaningful when teaching and how perceptions regarding disabilities evolve during a field experience that incorporates SL and critical reflection	50	young adults with disabilities (n = 24)	formal interviews (100), informal interviews, critical incident reports (312), formalized reflections (50), and direct observation (64).	Qualitative: To analyze the qualitative data, we took a grounded theory approach [73] and conducted content analysis of formal and informal interviews, critical incidents, formalized reflections, and field notes from observations.	3 distinct stages of development emerged illustrating students’ progression during SL: anticipation, familiarization, and commitment. The duration of each phase seemed to be unique to each student. Establishing and developing relationships were perceived as the most meaningful experience while communication and effective teaching strategies were most challenging. Critical reflection ensures that students advance from anticipation and familiarization to commitment, which constitutes change, not only in attitude and understanding, but in behaviour.

## Data Availability

Data available on request due to restrictions.
